# A review: hemocompatibility of magnetic nanoparticles and their regenerative medicine, cancer therapy, drug delivery, and bioimaging applications

**DOI:** 10.3389/fchem.2023.1249134

**Published:** 2023-08-29

**Authors:** Shirin Malehmir, Mohammad Ali Esmaili, M. Khaksary Mahabady, Ali Sobhani-Nasab, Amir Atapour, Mohammad Reza Ganjali, Ali Ghasemi, Amin Moradi Hasan-Abad

**Affiliations:** ^1^ Karaj Branch, Molecular Biology Research Center, Islamic Azad University, Tehran, Iran; ^2^ Department of Microbiology, Karaj Branch, Islamic Azad University, Karaj, Iran; ^3^ Department of Laboratory Sciences, Sirjan School of Medical Sciences, Sirjan, Iran; ^4^ Anatomical Sciences Research Center, Institute for Basic Sciences, Kashan University of Medical Sciences, Kashan, Iran; ^5^ Physiology Research Center, Institute for Basic Sciences, Kashan University of Medical Sciences, Kashan, Iran; ^6^ Department of Medical Biotechnology, School of Advanced Medical Sciences and Technologies, Shiraz University of Medical Sciences, Shiraz, Iran; ^7^ Center of Excellence in Electrochemistry, University of Tehran, Tehran, Iran; ^8^ Center of Excellence in Electrochemistry, School of Chemistry, College of Science, University of Tehran, Tehran, Iran; ^9^ Department of Biochemistry and Hematology, Faculty of Medicine, Semnan University of Medical Sciences, Semnan, Iran; ^10^ Autoimmune Diseases Research Center, Shahid Beheshti Hospital, Kashan University of Medical Sciences, Kashan, Iran

**Keywords:** magnetic nanoparticles, green method, hemocompatibility, cancer therapy, bioimaging, drug delivery

## Abstract

Nanoparticles have demonstrated noteworthy advancements in the management of various complex medical conditions, particularly cancer. In any case, these particles still harbor the potential to improve medicate conveyance to challenging, hard-to-reach loci. The interactions that occur between nanoparticles and red blood cells during their journey throughout the human body, despite exposure to blood, are still not fully understood. Assessment of the ability of nanoparticles to integrate with blood, characterized as nanoparticle compatibility, has been consistently overlooked and undervalued in its import. This review article investigates the effect of nanoparticles on red blood cells, while examining the compatibility of nanoparticles through the angle of hemolysis. This article discusses the main roles of erythrocytes and also provides an informed interpretation of several mechanisms involved in the interaction of nanoparticles and erythrocytes. Throughout the review, significant emphasis is attributed to the investigation of hemocompatibility studies concerning newly designed nanoparticles to promote their successful translation into clinical application. This review article examines the compatibility of magnetic nanoparticles in various fields, including regenerative medicine, cancer therapy, bioimaging, and drug delivery. Our results show that the chemical composition of the nanoparticle surface is a determining factor in hemocompatibility performance and interaction with blood cells. The surface properties of nanoparticles, namely surface charge, geometry, porosity, and surface functionalities of polymers or specific functional groups, represent key determinants of hemocompatibility.

## 1 Introduction

Nanoscience is one of the most important fields of modern science. Researchers in the field of biological sciences and healthcare are using nanotechnology to make substantial advances at the atomic and cellular levels (Tartaj et al., 2003; Faraji et al., 2010; Moradi Hasan-Abad et al., 2022). Because of their unusual size and physicochemical properties, nanoparticle compounds have various advantages. Magnetic nanoparticles (MNPs) are gaining popularity due to their unique superparamagnetism with controllable size, excessive chemical stability with more desirable surface, being surface functionalized with diverse molecules, and biocompatibility. External magnetic fields induce magnetic moments that are appropriate for immobilizing MNPs near physiological targets and diverse cell types (Ban et al., 2018; Cardoso et al., 2018; Hasan-Abad et al., 2022). Various forms of MNPs are now synthesized due to their widespread usage in engineering, healthcare, biotechnology, environmental science, and material science. In addition, MNPs are employed in lab-on-a-chip technologies, tissue engineering, magnetic hyperthermia therapy, antimicrobial compounds, magnetorelaxometry, drug delivery systems, and cell separation (Anik et al., 2021; Mohammadi et al., 2021).

Many studies and research projects on MNPs have lauded their importance in recent decades. However, there are still unknown areas that require more development. Magnetic nanostructures’ detrimental effects on cells and toxicity to physiological systems are currently a source of great worry. This review discusses green MNP synthesis strategies and their potential for biomedical applications. Furthermore, the features of MNPs, particularly those related to surface modification and biological applications, are discussed. Additionally, a dedicated segment has been curated to emphasize the significance of blood compatibility and potential concerns associated with the utilization of MNPs in various medical applications such as cancer treatment, bioimaging, drug delivery, and regenerative medicine.

## 2 Green synthesis of MNPs

**FIGURE 1:**
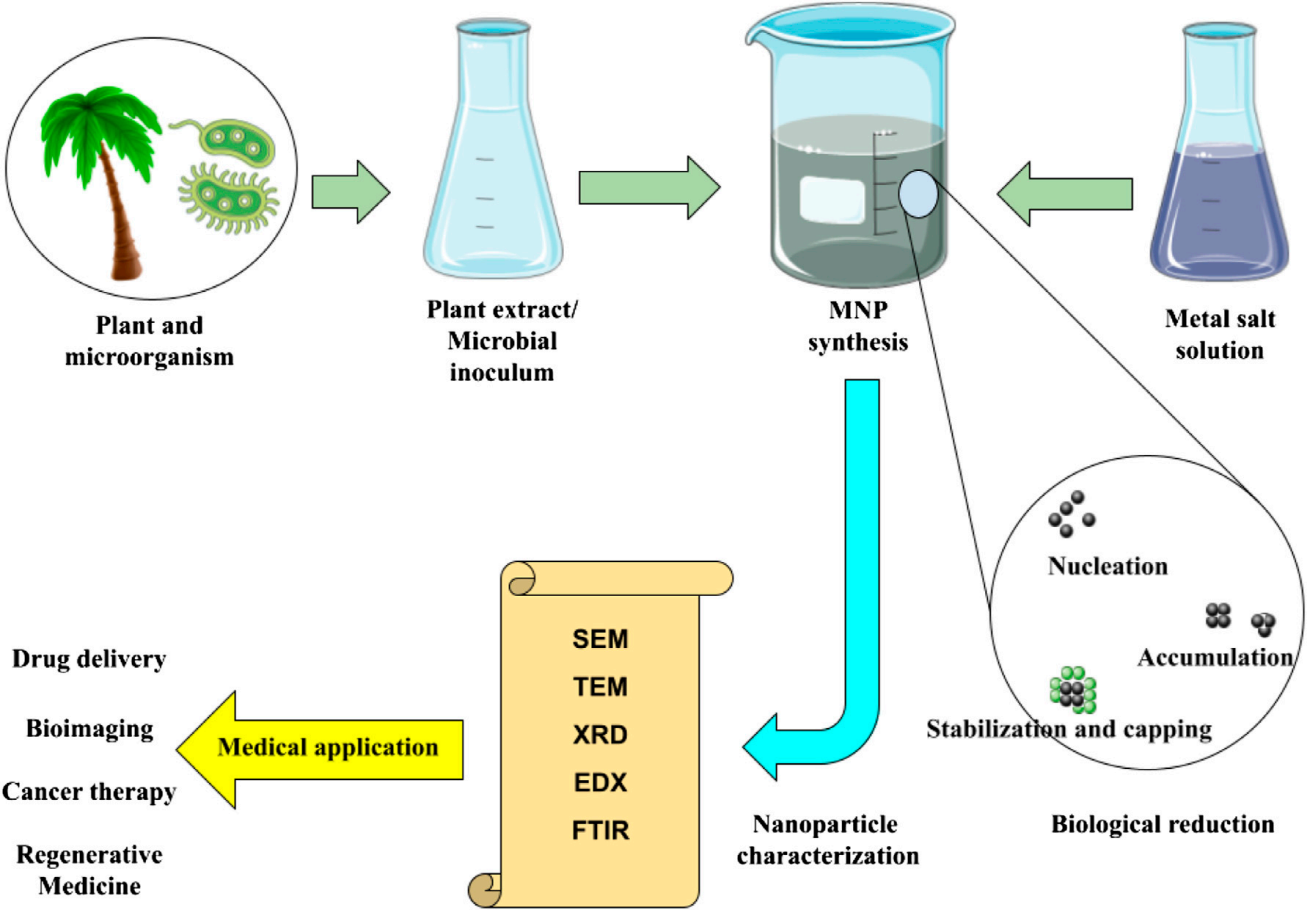
Green synthesis of magnetic nanoparticles, analytical methods, and biomedical applications.

In general, “green” synthesis in nanotechnology refers to the synthesis of nanostructures without the use of hazardous chemicals that produce toxic products. In other words, the green method is an environmentally friendly method of nanoparticle synthesis that causes no harm to human health and the environment. It is true that nanoparticles of the desired shape and size can be produced in large quantities by conventional methods. However, these techniques demand expensive production, convoluted processes, and out-of-date practices (Patra and Baek, 2014). Green methods offer a number of advantages over traditional chemical and physical methods, including an easy, straightforward manufacturing process, quick, economical production, and reduced waste generation. The bottom-up method of green biosynthesis of nanoparticles involves the assembly of metal atoms into clusters, which then develop into nanoparticles. During nanoparticle synthesis, biological compounds present in green materials can act as capping agents and reducing agents to stabilize nanoparticles (Figure 1). This allows for the control of the nanoparticles’ size and shape, which can be applied in a variety of ways. Only a green substrate and a metal salt (precursor) are required for the straightforward nanoparticle synthesis. During nanoparticle synthesis, various parameters such as metal salt concentration, green substrate concentration, reaction time, and solution temperature and pH can be changed to obtain the desired properties (Silva et al., 2015). When green biosynthesized MNPs are compared to physically synthesized MNPs, it is clear that they may have superior properties such as enhanced biocompatibility and biodegradability. Hence, apart from its non-toxic and friendly nature, the green material possesses a distinctive surface layer that facilitates precise drug delivery through the localization of Fe_3_O_4_ nanoparticles in designated regions. This characteristic renders it suitable for utilization in biomedical contexts. The utilization of environmentally sustainable materials in the manufacture of MNPs has the advantage of safe ingestion, hence reducing potential toxicity to the human body. This characteristic holds significant promise for various biological applications. MNPs provide the ability to selectively target specific tissues, organs, or tumors through the application of an external magnetic field. Additionally, MNPs can be utilized in the treatment with hyperthermia by mixing them with medicines, enzymes, or proteins that are heated in an alternating magnetic field (Reddy et al., 2012; Mahdavi et al., 2013).

## 3 Hemocompatibility of magnetic nanoparticles

Different medical applications of MNPs require direct contact with healthy tissue and blood. Poorly designed nanostructures can cause several problems, such as hemolysis or cytotoxicity. The ASTM F756 standard has been used to determine the hemolytic activity (% hemolysis) of different nanomaterials, and the percentage of hemolysis is calculated as the concentration of released hemoglobin divided by the concentration of total hemoglobin in the exposed red blood cells. In this standard, 0%–2% hemolysis is non-hemolytic, 2%–5% is slightly hemolytic, and more than 5% is hemolytic (Yedgar et al., 2022). Although today there are other methods such as atomic force microscopy and scanning electron microscopy, calcium ions also have been used as a reliable index to check the level of hemolysis (Stamopoulos et al., 2009; Lang et al., 2003; Romero and Hernández-Chinea, 2017; Kozlova et al., 2014). One of the strategies to improve the biological properties of MNPs is their functionalization with non-ionic surfactants and biocompatible polymers.

Numerous MNPs, particularly those based on iron oxide, have been studied for their hyperthermia potential. In one work, Hurtado et al. developed a composite of activated carbon and magnetite (AC/Fe_3_O_4_ composite), which was then heated in a low-frequency magnetic field. The heating results suggest that the 18 mg composite block may achieve a temperature of 45°C. The hemolysis test revealed that the chemical with a 4.7% hemolysis rate was non-hemolytic. The material, according to these findings, can be used in magnetic hyperthermia procedures for cancer treatment (Ríos-Hurtado et al., 2016). Another study described the development of new magnetic, blood-compatible nanoparticles based on naturally occurring chitosan and magnetite that could be used as biomaterials in nanobiomedicine. Rahman et al., in their study, prepared a magnetic nanocomposite that functionalized with the chitosan carboxy group (Fe_3_O_4_–CS–BTCDA). The hemolytic activity of the magnetic nanocomposites was tested, the degree of hemolysis rose with the increase in nanoparticle concentrations and incubation time, but the maximal hemolytic degree was only 3.2%. This hemolytic degree is less than the reported 5% acceptable limit of hemolysis for blood-contacting biomaterials. The finding of this research showed that the magnetic nanocomposites are hemocompatible and can be used in practical applications related to biological aspects (Rahman and Ochiai, 2018). Ceragenins (also known as cationic steroid antimicrobials (CSAs)) are tiny, synthetic compounds with antimicrobial and anticancer properties. Ceragenins is a good prospective antimicrobial drug because they mimic endogenous antibacterial peptides. Strong bactericidal action, on the other hand, has been linked to host cytotoxicity in some cases. Niemirowicz et al. synthesized MNP@CSA-13 to assess the antibacterial activity and biocompatibility of ceragenin, and all physicochemical and antibacterial experiments were carried out. MNP@CSA-13’s hemolytic activity was assessed by measuring hemoglobin release from human erythrocytes. The results of this investigation reveal that MNP@CSA-13 has no effect on erythrocyte membrane permeability (results indicate that MNP@CSA-13 caused ∼1% hemolysis) in the concentration range of 1–100 g/mL. This concentration of nanoparticles successfully kills planktonic *P. aeruginosa* as well as bacteria in mature biofilms. MNP@CSA-13 nanoparticles have the dual effect of lowering ceragenin’s hemolytic action while also improving antibacterial characteristics in bodily fluids. Furthermore, these functionalized MNPs illustrate the applicability of a platform for developing multitasking nanosystems that may be valuable in theranostic applications (Niemirowicz et al., 2015). MNPs were coated with a combination of polysorbate 80 and polyethylene glycol 3350 in one investigation conducted by Roacho-Pérez. The results showed that PEG 3350-Tween 80-coated MNPs with an average particle size of 119.2 nm were successfully produced. To assess the cytotoxicity of the nanoparticles, primary cultures of stem cells obtained from sheep adipose parenchyma were established. A hemolysis test uses a sample of red blood cells from a healthy donor. Interaction with mesenchymal stem cells has been proven to be non-cytotoxic at levels less than 1,000 g/mL. At concentrations less than 100 g/mL, the reaction in erythrocytes does not appear to be hemolysis (HR < 2%). Based on the results of this study’s *in vitro* testing, it was determined that Tween 80-PEG 3350-coated MNPs are safe in biological systems at low concentrations (Roacho-Pérez et al., 2020). Yalcin et al. constructed cobalt ferrite (CoFe_2_O_4_) nanostructures in one investigation. The production of a prominent black precipitate in the hemolytic activity test shows the formation of a heme–iron complex. The relatively large number of reactive oxygen species formed by cobalt ferrite nanostructures has been attributed to the poor hemolysis of human erythrocytes by CoFe_2_O_4_ nanoparticles at two concentrations (hemolysis ratios corresponding to 1.0 mg/mL and 5.0 mg/mL concentrations were 5.4% and 24.7%, respectively) (Yalcin et al., 2021). Karageorgou et al. used a radioactive isotope to label MNPs. In a hematological compatibility investigation, the new 68Ga–DPD–Fe_3_O_4_ based on magnetite Fe_3_O_4_ was evaluated against human RBC. At the micro- and nanoscales, atomic force microscopy and optical microscopy were utilized to assess the morphological and geometric properties of entire cells. The findings of this study demonstrate that 68Ga–DPD–Fe_3_O_4_ has no effect on the general form and size characteristics of RBCs at the microscopic level, but RBCs exhibit two primary findings at the nanoscopic level, holes and ulcer-like abnormalities (Karageorgou et al., 2020). Liao et al. created Fe_3_O_4_@Alg and Fe_3_O_4_@Alg–GA by combining Fe_3_O_4_ with alginate and D-galactosamine to develop a ferrofluidic formulation capable of selectively targeting tumors and boosting temperature while staying biocompatible. Because of improved cellular absorption, Fe_3_O_4_@Alg–GA nanoparticles displayed good thermogenesis in a human hepatocellular carcinoma (HepG2) cell line. The anti-hemolytic activity and hemocompatibility of the generated nanoparticles were evaluated using a red blood cell hemolysis assay. Both Fe_3_O_4_@Alg nanoparticles and Fe_3_O_4_@Alg–GA nanoparticles displayed excellent blood compatibility, comparable to PBS, indicating that our Fe_3_O_4_@Alg and Fe_3_O_4_@Alg–GA nanoparticles lacked hemolytic activity. The results presented in this study show that these nanoparticles are suitable for *in vivo* intravenous delivery (Liao et al., 2015). Ineffective cellular targeting has hampered MNP-induced hyperthermia therapy. pH-responsive charge-conversional MNPs can improve selective cellular absorption in acidic cells such as malignancies by sensing extracellular acidity via charge change. Rahman et al. created new pH-induced charge-conversional, superparamagnetic, and single-cored Fe_3_O_4_ nanocomposite particles covered with N-itaconylated chitosan (NICS) and cross-linked with ethylene glycol diglycidyl ether (EGDE) (Fe_3_O_4_–NICS–EGDE). To improve buffering capacity, aqueous stability, and pH sensitivity, the surface of the Fe_3_O_4_–NICS–EGDE nanocomposite particles was changed with ethanolamine (EA) through aza-Michael addition. In physiological environments, the proposed Fe_3_O_4_–NICS–EGDE–EA nanocomposite particles demonstrated pH-dependent charge-conversional characteristics, colloidal stability, and high hemocompatibility. The degree of hemolysis grew as the nanocomposite concentration climbed and the pH decreased, but it remained below 2%. As a result, the composite particles are ideal for magnetically induced and targeted cellular thermotherapeutic applications (Rahman et al., 2018). In another investigation, Muzquiz-Ramos et al. created Fe_3_O_4_@CaSiO_3_ nanostructures with sizes ranging from 5 to 10 nm by covering Fe_3_O_4_ nanostructures with CaSiO_3_ and then immersing them in simulated bodily fluids. A hemolysis test based on hemoglobin release revealed that none of the samples displayed hemolysis up to 3 mg/mL, indicating no damage to the red blood cell membrane (the hemolytic test findings revealed that the HRs of the samples were less than 2%). These bioactive, hemocompatible, and superparamagnetic particles may have use in the hyperthermia therapy of bone cancer (Muzquiz-Ramos et al., 2012). Szekeres et al. created polyacid–covered core–shell iron oxide nanoparticles for prospective use in biomedicine, with a focus on theranostics—MRI, magnetic hyperthermia, and magnetic drug targeting. The MNPs (PGA@MNPs) were tested for hemocompatibility in blood, sedimentation rate, blood smear, and blood cell viability experiments, as well as antioxidant capacity in Jurkat cells in the presence of H_2_O_2_ as reactive oxygen species. There was no evidence of nanoparticles interacting with entire blood cells. Furthermore, the PGA@MNPs considerably reduced oxidative stress mediated by H_2_O_2_, validating previous MTT findings, namely, the enhancement of cell viability in their presence. *In vitro* experiments indicated that PGA@MNPs are not only biocompatible but also bioactive. Preliminary tests demonstrated that the nanoparticles are very effective MRI and magnetic hyperthermia agents (Szekeres et al., 2015). Wang et al. created PAMNPs (polyampholyte-coated polyacrylic acid PAA-co-3-diethylaminopropylamine MNPs) as contrast agents for magnetic resonance imaging. The contrast agents used in high-resolution magnetic resonance angiography must be biocompatible. The results demonstrated that MNPs at 0.138 mM exhibited hematological compatibility comparable to the negative control and, consequently, had no significant effect on blood vessels. The test results showed that hemolysis rates were less than 5% for all PAMNP doses tested. Taken together, these results show that magnetite nanoparticles have enormous promise as safe contrast agents for magnetic resonance imaging (Wang et al., 2015). Li et al. created Fe_3_O_4_@Au nanostructures with a composite core that will be studied for their potential use in tumor hyperthermia. A cytotoxicity test and a hemolysis test were carried out. In a cytotoxicity study, the substance’s toxicity to the mouse fibroblast cell line (L-929) was determined to be Class I, suggesting that it is not cytotoxic. The Fe3O4@Au composite hemolysis rate ranged from 0.278% to 0.232%, which was significantly lower than 5% (Li et al., 2011).

**FIGURE 2:**
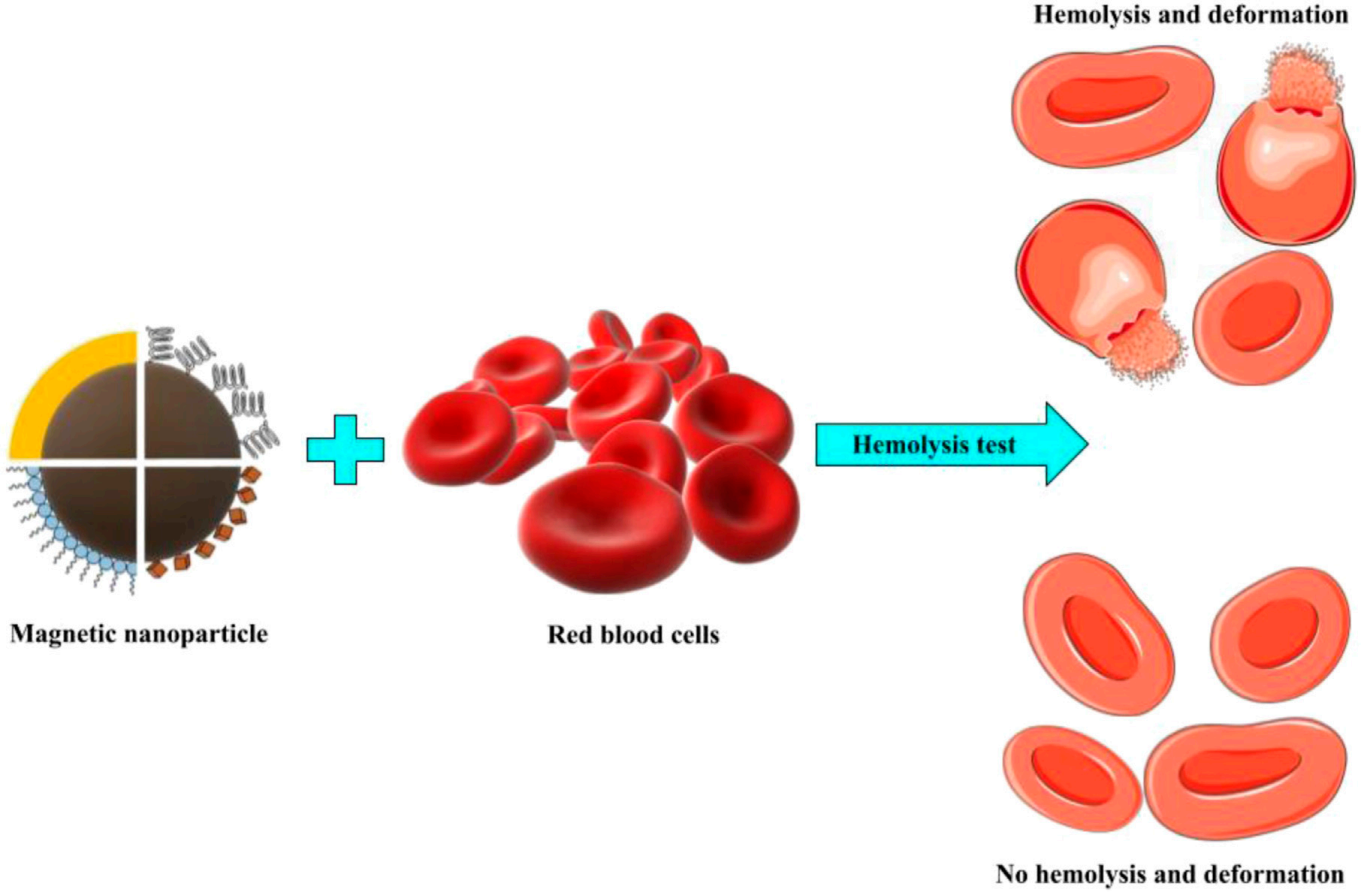
Hemolysis is shown schematically where magnetic nanoparticles interact with red blood cells.

These findings show that the chemical composition of the MNP surface has a proportional effect on hematopoietic fitness and contact with blood cells (Figure 2). The geometry, surface charge, surface functionalities, and porosity of polymers or specific functional groups are among the most important surface attributes of nanoparticles in terms of hemocompatibility and should be considered when evaluating nanoparticle hemocompatibility.

### 3.1 Hemocompatibility of magnetite nanoparticles in regenerative medicine

The field of regenerative medicine, which is characterized by its innovative nature, aims to reinstate the proper functioning of damaged cells, organs, and tissues through repair mechanisms. The advancement of effective therapeutic stem cell therapy requires interdisciplinary collaboration among several scientific fields. Nanotechnology facilitates the conversion of fundamental scientific inquiry into practical applications in various domains. The utilization of MNPs has proven to be effective in the labeling, tracking, and activation of stem cells, hence offering a very efficient means of monitoring their behavior both *in vitro* and *in vivo*. The issue of safety is a persistent concern, highlighting the importance of conducting research on toxicity. Over time, the presence of any form of toxicity reduces the efficacy of the treatment. The objective of this section is to present a concise overview of contemporary research pertaining to this subject matter, with particular emphasis on hemolysis assays that are commonly employed (Markides et al., 2012).

Polymer composites that respond to magnetic fields have enormous promise for use in a wide range of biomedical applications. Lalegul-ulker et al. developed composite biomaterials made of superparamagnetic iron oxide (SPION) nanoparticles and silk fibroin (SF nanofiber magnetic). *In vitro* indirect cytotoxicity and *in vitro* hemocompatibility studies are performed on these composites. The percentage *in vitro* hemolysis results in the experimental groups were close to each other and all were 0.5%. Overall, the findings revealed that all SF nanofiber magnetic materials were hemocompatible and harmless to fresh blood. Overall, the findings suggested that this nanofiber could be employed in tissue engineering as a magnetically reactive cytocompatible scaffold, to stimulate stem cell differentiation *in vitro*, and/or as biosensors (Lalegül-Ülker et al., 2019). Michalicha et al. created and studied functional hydrogels made from curdlan, a naturally occurring polymer, and supplemented with Fe_3_O_4_ nanoparticles or Fe_3_O_4_-based heterostructures and poly (L-DOPA) (PLD). Some of the modified hydrogels created were non-toxic and moderately hemocompatible and showed strong antibacterial potential as well as the ability to convert energy through heat generation. As a result, the proposed hydrogels may have uses in temperature-controlled regenerative processes as well as oncology therapy as a matrix of improved functionality for a variety of medicinal purposes. PLD in the curdlan hydrogel network reduced NP release while somewhat increasing the hydrogel’s hemolytic capabilities. This investigation found that blood hemolysis did not surpass 5% of the positive control in cases of blood contact with all samples except CR–PLD–Fe_3_O_4_–Ag, showing a lack of hemolytic impact. Only the CR–PLD–Fe_3_O_4_–Ag hydrogel showed 13% hemolysis, indicating that the sort of prospective application for this hydrogel should be carefully considered, excluding components in frequent contact with blood (Michalicha et al., 2023). Thrombosis-related diseases kill a lot of people and pose a severe threat to health and life. Nattokinase (NK), which has a high thrombolytic effect, can be used to treat thrombotic diseases. In one investigation, Liu et al. produced NK-conjugated MNPs (NK-MNPs) to specifically deliver NK to the thrombus site. MNPs composed of FeO, carboxymethyl chitosan, and sodium alginate were created to encapsulate NK. *In vitro* thrombolysis experiments have proven the ability of NK-MNPs to perform sustained-release thrombolysis. NK-MNPs offer potential for increased thrombolytic efficacy with prolonged release and magnetic targeting and have been shown in a hemolysis experiment to have a hemolysis rate of less than 5% (Liu et al., 2021).

In one work, Lalegul-ulker et al. created silica-polyaniline-coated iron oxide nanocomposites (Si-MNPs/PANI) for biological applications. Si-MNP/PANI particles (SGT) and nanotubes (SPNT) were created by altering synthesis parameters such as acid concentration and mixing technique. The biological properties were assessed using an indirect *in vitro* cytotoxicity test and an *in vitro* blood compatibility test. In general, all nanocomposites with a dose of 10 mg mL^−1^ have a hemolysis value less than 5% (typically 2%–4%), demonstrating the safety of this dose. The findings reveal that the *in vitro* cytocompatibility and blood compatibility fractions of SGT and SPNT fluctuate in a dependent manner, indicating that they are used in specialized applications, such as regenerative medicine (Lalegül-Ülker and Elçin, 2021). Bioactive extracellular matrix-based materials mimic the complicated structure and composition of natural tissues. The decellularized cancellous bone matrix (DBM) has the ability to speed up regeneration and direct bone growth. Low-frequency pulsed electromagnetic field (LF-PEMF), on the other hand, has been proven to boost bone defect regeneration ability. In a previous study, Parmaksiz et al. investigated whether DBMMNP and LF-PEMF could be utilized to cure critical-size bone lesions. DBMMNP was produced and a hematological compatibility test was performed. *In vitro* hemolysis experiments revealed that DBMMNP grafts were non-hemolytic in general, with values of 5.0%. The hemolytic effect increased slightly when the MNP level in DBM grew from 1% to 5% MNP (Parmaksiz et al., 2021). Stem cells play a role in physiological processes like as angiogenesis following ischemia and vascular regeneration, which help to regenerate cardiac defects or repair vascular damage. However, the therapeutic efficacy of stem cell transplantation is frequently limited by poor collection of systemically delivered cells, resulting in low cell counts in sick locations. Fe_3_O_4_ PEG–CD34 magnetic nanoparticles coated with an anti-CD34 antibody were demonstrated to exhibit a substantial affinity for stem cells in one study led by Chen. Nanoparticles must be compatible with blood in order to be administered intravenously. The measured hemolysis rate for anti-CD34-coated MNPs was only 0.13%, demonstrating the absence of hemolysis. The results of a PTT test for hemolysis rate and activated partial thromboplastin time showed that these nanoparticles are safe for usage in the bloodstream (Chen et al., 2013). In one work, Xin Liu et al. created a multifunctional silk fibroin-based composite scaffold. Despite its great biological qualities and relatively short disintegration, silk fibroin’s utility for long-distance vascular defect repair is severely limited. A better infiltration strategy resulted in the creation of numerous new magnetic SF scaffolds (MSFCs). MSFCs were discovered to have higher crystallinity, mechanical strength, and magnetocaloric properties than SF platforms (SFC), which was attributed to the levelheaded presentation of iron-based attractive nanostructures. This study’s results, which examined the rate of hemolysis, show that SFC, MSFC10, MSFC50, and MSFC100 have outstanding blood compatibility (Liu et al., 2022). Recent research studies have presented encouraging findings concerning the application of extracorporeal magnetic separation-based blood purification for the swift and targeted elimination of disease-causing substances from whole blood. This technique has demonstrated the ability to remove cells, bacteria, and high-molecular-weight compounds from blood within a matter of minutes. As a result, it presents innovative treatment approaches for addressing intoxications and bloodstream infections (Herrmann et al., 2015; Gao et al., 2022). A new fabrication procedure for magnetic nanocomposites mixed with heparin and chitosan (MNPs@CS and MNPs@CS@Hep) as blood purifiers to remove LDL low-density lipoproteins from plasma is presented in a study by Jinghua Li et al. Because it contains a magnetic core, the absorbent material can be easily detached for reuse using an external magnet, and the plasma level of LDL dropped by 67.3% after 2 h of blood perfusion. The hemolysis rate of MNPs@CS and MNPs@CS@Hep is less than 5% of clinical guidelines, according to the blood compatibility test (Li et al., 2014). Magnetically assisted hemodialysis is a modification of conventional hemodialysis based on the circulation of ferromagnetic nanoparticle-targeted binding substance conjugates (FN-TBS Cs) in the patient’s bloodstream and their eventual elimination using a ’magnetic dialyzer’. Stamopoulos et al. investigated the biocompatibility of Fe_3_O_4_FNs and Fe_3_O_4_-bovine serum albumin Cs (Fe_3_O_4_–BSA Cs) with blood cells *in vitro*. Atomic force microscopy (AFM) and optical microscopy (OM) enabled the nanometer- and micrometer-scale examination of blood cells, respectively. Both AFM and OM revealed no qualitative alterations in the overall shape of blood cells. Extremely rarely did Fe_3_O_4_FNs or Fe_3_O_4_–BSA Cs bind to the surface of RBCs or become internalized by WBCs. Detailed examination with OM demonstrated that neither crude Fe_3_O_4_FNs nor Fe_3_O_4_–BSA Cs initiate or promote impaired platelet coagulation. The statistical analysis of the acquired AFM images of RBC surfaces revealed that the mean surface roughness of RBCs matured with Fe_3_O_4_FNs or Fe_3_O_4_–BSA Cs was identical to that of reference RBCs (Stamopoulos et al., 2008). Zhenqiang et al. created a new type of imidazolium-based ionic liquid with antibacterial activity and polydopamine coating was used as a hemocompatible platform to immobilize ionic liquids on Fe_3_O_4_ nanoparticles, resulting in hemocompatible magnetic particles (Fe_3_O_4_@PDA-IL). The hemolysis ratio of the Fe_3_O_4_@PDA-IL particles at 1 mg/mL concentration was substantially lower than the warning level of 5%, and the erythrocytes remained intact after incubating with the particles. The magnetic particles were hemocompatible and effective in removing clinically significant pathogens from human whole blood, including Methicillin-resistant *S. aureus*, *P. aeruginosa*, *E. coli*, and *S. aureus*, which are the most common pathogens in bloodstream infections. Furthermore, the Fe_3_O_4_@PDA-IL particles were able to eliminate bacterial endotoxins from blood, preventing further deterioration of sepsis (Shi et al., 2020). The number of circulating tumor cells (CTCs) has been linked to a poor prognosis in various forms of cancer. Doswald conducted one study with the goal of designing and testing new MNPs to purify whole blood from CTCs. Antifouling and separation capabilities of novel magnetic carbon-coated cobalt (C/Co) nanoparticles linked with anti-epithelial cell adhesion molecule (EpCAM) antibodies were determined. The novel C/Co nanoparticles demonstrated remarkable separation and antifouling properties. Through an anti-EpCAM antibody interaction, they effectively eliminated tumor cells given to healthy patients’ blood samples. Other blood components, such as lymphocytes and the coagulation system, were not affected by the nanoparticles (Doswald et al., 2022).

### 3.2 Hemocompatibility of MNPs in cancer therapy

Cancer is an uncontrollable cell growth and proliferation which finally resulted in the spread of cancerous cells to other parts of the body (metastasis phase). Surgery, chemotherapy, and radiotherapy are conventional treatments for cancers, and their combination and transpositions depend on the type of the cancer. However, these treatments have serious side effects and also have not adequate efficacy for the treatment of cancers in metastatic stages (Hasan-Abad et al., 2019; Keyvani et al., 2022). MNPs could be helpful for treatment of these types of cancers (Table 1). Usually, MNPs are used in combination with active substances and biocompatible materials to improve their stability and for the prevention of agglomeration and reduction of the resistance risk. Thus, MNPs could be promising therapeutic targets for oncological studies. The biocompatibility and possible toxicity of apatite-coated MNPs were examined by Elia Martha et al. They began by looking into the biocompatibility of these MNPs with RBCs *in vitro*. Their findings revealed no hemolytic activity at doses less than 3 mg/mL. Furthermore, they administered these MNPs into Balb/c mice at doses ranging from 100 to 2,500 mg/kg, and the results indicated normal liver and kidney function with no significant changes in body weight or liver iron burden. Apatite-coated MNPs with a size of 2–8 nm show no toxicity *in vivo* and no hemolytic activity *in vitro* (Múzquiz-Ramos et al., 2013). Zhang et al. investigated the biocompatibility of Fe_3_O_4_ MNPs *in vitro* and *in vivo*. An MTT assay and hemolysis test were used to investigate cytotoxicity and blood toxicity, respectively. They also injected these MNPs into KM mice to determine the LD_50_. The results demonstrated that L929 cell lines were cytotoxic to varying degrees. Furthermore, no hemolysis or genotoxicity was observed, and the LD_50_ was 7.57 g/kg. Taken together, nanosized Fe_3_O_4_ is a good material for tumor hyperthermia because of its great biocompatibility (Zhang and Du, 2006). Magnetic drug targeting, magnetic hyperthermia, and theranostics, MRI, could all benefit from polyacid-covered core–shell iron oxide nanoparticles. Szekeres et al. investigated PGA@MNP, a gallic acid shell-coated MNP, in terms of hemocompatibility, sedimentation rate, and antioxidant capacity in the presence of H_2_O_2_ as reactive oxygen species, blood vitality, and blood smear. They discovered that these MNPs may dramatically lower oxidative stress, which was linked to an increase in cell viability in the MTT experiment. Furthermore, there is no contact of these components with whole blood, and *in vitro* investigations demonstrated its bioactivity. The r2 relaxivity and specific absorption rate (SAR) of PGA@MNPs are similarly significant (387 mM-1s-1 and 11 W/g magnetite, respectively) (Szekeres et al., 2015). Daunorubicin (DNR) is a chemotherapeutic drug used to treat acute lymphocytic leukemia (ALL) and acute myeloid leukemia (AML). Wu et al. studied the hematologic and genotoxicity of Fe_3_O_4_ MNPs coupled with DNR (Fe_3_O_4_/DNR), as well as their effectiveness in malignancies. The LD_50_ was determined by injecting these MNPs intra-peritoneally into mice and testing the drug’s liver and kidney damage. They discovered that the hemolysis rate of this combination varies with dose, ranging from 2.908% to 2.415%. The predicted LD_50_ of these nanoparticles was 1,009.71 mg/kg, and no significant weight differences were observed across treatment groups at 3 days following injection. There were no hazardous effects such as convulsions, irritability, respiratory failure, or paralysis. BUN, liver enzymes, and renal function did not differ significantly between the control and DNR groups. As a result, Fe_3_O_4_/DNR is a safe and biocompatible nanoparticle that could be employed to treat hematopoietic malignancies (Wu et al., 2010). PGA@MNPs are made from iron salts, amino cellulose, and polyethylene glycol and are utilized to induce apoptosis (through niclosamide) and hyperthermia for tumor eradication. Ahmad et al. investigated the hemocompatibility and theranostic potential of PGA@MNPs. They evaluated the impact of these NPs on HCT116 cells and discovered that 200 g/mL blank NP is cyto- and hemocompatible. Furthermore, niclosamide-loaded functionalized magnetic nanoparticles (NFMNPs) would be seven times more effective than free niclosamide in killing colorectal cancer cells (CRC) cells. NFMNPs have the ability to trigger apoptosis and, when exposed to an alternating magnetic field (AMF), produce more niclosamide, which is associated with increased cell death. As a result, hyperbranched polymer-functionalized MNPs could be a promising method for niclosamide administration and cancer treatment (Ahmad et al., 2019). Current colorectal cancer treatment includes oxaliplatin (Oxa) combined with biological agents. However, the administration of this medicine causes adverse effects that limit its use in patients, necessitating the development of new methods for targeted chemotherapy. MamC-mediated biomimetic magnetic nanoparticles combined with Oxa (Oxa-BMNPs) have previously been shown to significantly reduce the IC50 when compared to soluble Oxa. Their robust contact with macrophages, however, demonstrated toxicity and the likelihood of aggregation. Oxa-BMNP nanoassemblies were encased in phosphatidylcholine unilamellar liposomes (both PEGylated and non-PEGylated) in the current investigation by Garcia-Pinel et al. Their findings show that adding a lipid cover as well as further PEGylating increases the biocompatibility and cellular uptake of Oxa-BMNP nanoassemblies without significantly lowering their lethal effect in colon cancer cells. The PEGylated magnetoliposome nanoformulation, in particular, reduced hemolysis from 5% to 2%, making them hematocompatible, reduced red blood cell agglutination, and eradicated toxicity in white blood cells. This paper marks a significant advancement in this field because it outlines the creation of one of the very few extant nanoformulations that could be employed for local chemotherapy to treat CRC (Garcia-Pinel et al., 2020). Bladder cancer is a prevalent malignancy with a high death rate when treated with chemotherapy drugs like doxorubicin (DOX). Treatment of this form of malignancy presents various difficulties, including limited urothelium permeability and urinary voding. Intravesical drug delivery has been shown in studies to lessen agent side effects, reduce drug requirements, and boost drug concentration at the lesion site. Lv et al. studied multi-responsive liposomes for doxorubicin administration with a photothermal effect. They created FA-TMLs@MNPs–GNRs–DOX by encapsulating MNPs, DOX, and gold nanorods (GNRs) in folate-modified thermosensitive liposomes. FA-TMLs@MNPs–GNRs–DOX also bind to folate-receptor-positive cells and were lethal to bladder carcinoma cells. With FA-TMLs@MNPsEven at doses of up to 2,000 g/mL, GNRs did not trigger hemolysis. FA-TMLs@MNPs–GNRs–DOX could be employed as a combined drug delivery system for bladder cancer (Lv et al., 2021). Similarly, Li et al. evaluated the impact of FA–Fe_3_O_4_@nGO–DOX MNPs on cancer cells. These particles were treated with nanoscale graphene oxide (nGO) and Fe_3_O_4_ connected to folic acid and DOX and MGC-803 cells (gastric cancer cell line). The results demonstrated that MGC-803 selectively reabsorbed these MNPs, with a relative viability of roughly 90% after 48 h of therapy. Based on hemolysis tests, these particles demonstrated low toxicity and high anticancer activity, according to histological examination. As a result, these MNPs could be employed in medication delivery systems (Li et al., 2018). Hepatocellular carcinoma is the most prevalent form of primary liver cancer, and it usually originates in the context of chronic liver disorders such as hepatitis B or C infection. HCC could be diagnosed and treated using photothermal ablation and theranostic agents. Melanin pigments are very biocompatible and biodegradable. Chen et al. created melanin-based MNPs conjugated with the near-infrared (NIR) dye IR820 and encapsulated in PEG (IR820–PEG-MNPs), which boosts water solubility and photoacoustic (PA) and photothermal treatment performance. The results revealed strong PA and T1-weighted MRI signals, as well as a highly efficient photothermal impact. These particles may identify lesions as small as 1.8 mm in diameter. Under the direction of PA/MR imaging, IR820–PEG-MNPs demonstrated a high anticancer efficacy with a photothermal conversion efficiency of about 40.2% in an HCC animal model. Consistent with this, even at the highest concentration of 2.5 mg/mL, the hemolysis ratio was less than 4.8%, indicating that the IR820–PEG-MNPs are hemocompatible and can be injected intravenously for *in vivo* biological imaging. Overall, melanin-based medicines are useful for the early identification and treatment of HCC (Chen et al., 2020). Ceragenin possesses anticancer potential; nonetheless, its low hemocompatibility and high concentration for the desired effect against cancer have limited their utilization. Piktel et al. coupled or bonded CSA-131 with aminosilane-modified iron oxide-based MNPs (CSA-131+MNP and MNP@CSA-131) to improve therapeutic benefits while decreasing toxicity. These particles were used to treat lung and colon cancer cell lines, and the results demonstrated a considerable increase in the efficacy of CSA-131 action against cancer cells. Other findings of this study included a decrease in effective ceragenin dose from 1.17 to 34.57 times, indicating a positive interaction between CSA-131 and MNPs. CSA-131+MNP and MNP@CSA-131 both triggered apoptosis and reduced hemolytic activity by around six times when compared to free CSA-131. At a dose of 20 g/mL, MNP@CSA-131 and CSA-131+MNP induce hemoglobin release from only 3.07 to 3.57% and 1.47 to 0.79% of red blood cells, respectively, whereas CSA-131 alone damages 34.04 to 10.12% of RBCs. As a result, CSA-containing MNPs are prospective therapeutic agents for colon and lung cancer (Piktel et al., 2020). Dorjsuren et al. created cetuximab-coated thermosensitive liposomes filled with MNPs and doxorubicin for improved breast cancer treatment efficiency. They created acid-coated iron oxide magnetic nanoparticles (CMNPs) contained in liposomes (TLS). Furthermore, these particles were coated with cetuximab (CET), an anti-EGFR antibody, to preferentially target EGFR-expressing cells. Because the liposomes were thermosensitive, breast cancer cells demonstrated decreased viability after treatment with CET–DOX–CMNP–TSLs and the addition of photothermal therapy. Furthermore, hemolysis tests revealed good hemocompatibility, indicating that they were suitable for systemic administration (Dorjsuren et al., 2020). Guo et al. created Fe-polyhydroxy coordination polymer nanostructures (TA–Fe@MNPs) to improve tumor targeting. The TA–Fe@MNPs were created employing a TA-Fe(III) polymer, mannose (M) as a targeting factor, and carriers polyethyleneimine (PEI) and bovine serum albumin (BSA). TA–Fe@MNPs demonstrated good biocompatibility, an average size of 125 nm, and low normal cell toxicity. Furthermore, the TA–Fe@MNPs demonstrated excellent photothermal performance with a pH-sensitive Fenton-like response that was increased by glutathione response. Due to their M alteration and carrier materials, these particles persisted at the tumor site for up to 36 h with a proper photothermal action and gradual removal from the tumor site with a single injection. When the TA–Fe@MNP concentration was 100 μg mL^−1^, the hemolysis ratio increased to 1.24%. However, these results were less than the allowable maximum of 5%. An *in vivo* investigation validated their safety and efficacy in tumor ablation, offering fresh information concerning the use of photothermal agents in clinical trials (Guo et al., 2021).

**TABLE 1:** Magnetic nanoparticles with various sizes, production methods, hemolysis rates, and cytotoxicity.

Magnetic nanoparticles	Synthesis method	Nanoparticles size	Hemolysis rate	Cytotoxicity	References
Fe_3_O_4_–triethylene glycol and Fe_3_O_4_–tresyl chloride	Oxidation precipitation methods	10–30 nm	5.92% and 3.94%, respectively	-	Kempe and Kempe (2010)
Apatite-coated magnetite nanoparticles	Chemical co-precipitation technique	8 ± 2 nm	Lower than 2%	100 to 2,500 mg/kg did not cause apparent toxicity in Balb/c mice	Múzquiz-Ramos et al. (2013)
MNP@PEG-Cur	Co-precipitation method	24.33–34.24 nm	Less than 5%	Up to 100 μg/mL indicating the biocompatibility	Ayubi et al. (2019)
MNP + polylactide MNP + polysaccharide MNP + albumin	Co-precipitation method	122 ± 38 nm, 66 ± 19 nm, 192 ± 58 nm	31%–41%	-	Toropova et al. (2021)
Green synthesized magnetic iron oxide	Co-precipitation procedure	32–100 nm	14%	In AMN3 cells IC50 = 445 μg/mL	Tawfeeq (2017)
PEG–PCCL-MNP	Co-precipitation method	79.6 ± 0.945 nm	Slight hemolysis (3%)	-	Li et al. (2017)
GaFeO_3_	Sol–gel method	Between 15 and 20 nm	Lower than 2%	-	Sánchez et al. (2014)
Activated carbon/Fe_3_O_4_	Co-precipitation method	Between 9 and 14 nm	4.7% hemolysis	-	Ríos-Hurtado et al. (2016)
Magnetite–chitosan (Fe_3_O_4_–CS) Fe_3_O_4_–CS–BTCDA	Chemical co-precipitation technique	30 nm	3.2%	-	Rahman and Ochiai (2018)
Ceragenin-coated MNPs	Massart’s procedure	14 nm ± 2 nm	∼1% hemolysis	-	Niemirowicz et al. (2015)
Chit MNPs	-	292 to 127 nm	Lower than 5%	IC50 = above 2,500 and 3,000 μg/mL	Jesus et al. (2020)
CoFe_2_O_4_ nanoparticles	Co-precipitation methods	30–50 nm	5.4%	-	Yalcin et al. (2021)
Magnetite nanoparticles coated with PEG 3350-Tween 80	Chemical co-precipitation	119.2 nm	2% of hemolysis	-	Roacho-Pérez et al. (2020)
FeCl_2_:FeCl_3_ of 2:1 and 3:2, magnetite nanoparticles	Co-precipitation method	Between 8 and 12 nm	Lower than 2%	-	Macías-Martínez et al. (2016)
Fe_3_O_4_@Alg–GA nanoparticles	Co-precipitation method	128 ± 18.9	Nanoparticles did not have any hemolytic activity	-	Liao et al. (2015)
Maghemite nanoparticles	Chemical co-precipitation technique	12.5 nm	Lower than 2%	-	Múzquiz-Ramos et al. (2015)
Cobalt ferrite (CoFe_2_O_4_)	Co-precipitation	170 nm–250 nm	Over 200%	-	Drašler et al. (2014)
Fe_3_O_4_–NICS–EGDE–EA	Chemical co-precipitation-coating technique	300 nm	Below 2%	-	Rahman et al. (2018)
Fe_3_O_4_ nanoparticle	Co-precipitation technique	5–10 nm	Lower than 2%	-	Muzquiz-Ramos et al. (2012)
Met–MNPs and Met–MNPs–Amp		193.13 ± 6.8 nm and 218.53 ± 13.4 nm		IC50 at 50 μg/mL against both *A. castellanii* trophozoites and cysts	Abdelnasir et al. (2020)

### 3.3 Hemocompatibility of MNPs in bioimaging

Nanomaterials’ biocompatibility and physicochemical properties make them ideal for bioimaging and disease detection. Gold nanoparticles, magnetic nanoparticles, and carbon nanotubes are among the most active bioimaging research fields. Hocaoglu et al. studied the cyto- and biocompatibility of theranostic magnetic hybrid nanoparticles (Ag_2_S–Fe_3_O_4_). They created hybrid nanoparticles out of Ag_2_S quantum dots (QDs) and superparamagnetic iron oxide (SPION) using ligand exchange processes. Because these nanoparticles emit between 840 and 912 nm, they can be used as effective optical probes in medical imaging by reducing cell autofluorescence. They also demonstrated a high magnetic response as well as good cyto- and biocompatibility. The high cell cytotoxicity was observed after incubating HeLa and NIH/3T3 cell lines with these nanoparticles in a magnetic field. Within the examined dose range (1 g/mL to 100 g/mL), these two hybrid nanoparticles generated no significant hemolysis or changes in the size or count of red blood cells. Overall, their findings suggested that hybrid nanoparticles could be valuable for disease imaging and treatment (Hocaoglu et al., 2015).

PEGylated L-arginine-modified iron oxide nanostructures (PEG-Arg@IONPs) were produced and employed as MRI contrast in BALB/c mice by Nosrati et al. In addition, blood adaptability of PEG-Arg@IONPs was determined using an *in vitro* hemolysis assay. PEG-Arg@IONPs had no effect on blood HRBCs. It was determined that the hemolytic activity was less than 2.8%. A high-intensity MR imaging of the kidneys revealed the emission of these nanoparticles. Furthermore, signal intensity changes verified the existence of these particles in the liver, heart, and spleen, making these nanoparticles suitable T2 contrast agents for diagnosis (Nosrati et al., 2019). The high r2 relativity of Fe_3_O_4_ nanoparticles makes them an excellent contrast agent in MRI imaging. However, their poor targeting ability and colloidal stability have limited their application. Zhang et al. created an HA-stabilized Fe_3_O_4_ nanoparticle (Fe_3_O_4_@HA). HA has a high affinity for the CD_44_ receptor, which is expressed in a variety of malignancies. Fe_3_O_4_@HA nanoparticles exhibited an average size of 20 nm, excellent colloidal stability in water, and outstanding cyto- and hemocompatibility. They discovered that the percentage of hemolysis is almost negligible (less than 3%) under the current Fe concentrations by comparing absorbance at 541 nm. As a result, they determined that our Fe_3_O_4_@HA nanoparticles were hemocompatible, warranting further *in vivo* examination. They could also target CD_44_ receptor-overexpressing HeLa cells and be employed as a contrast agent for MRI *in vitro* and *in vivo*. As a result, these nanoparticles may increase the contrast of lesions in MRI (Zhang W. et al., 2022). Unterweager et al. coated SPIONs with dextran in order to employ them as an intravenous iron contrast agent in MRI. They looked at complement activation-related pseudoallergy (CARPA) for hypersensitive allergic responses after the treatment. SPIONdex demonstrated low hemolysis, leukocyte and platelet activity, and plasma coagulation *in vitro*. Furthermore, up to 5 mg Fe/kg SPIONdex did not elicit CAPRA in pigs. After 15 min of injection, the liver signal strength decreased and remained detectable for up to 24 h. This study concluded that SPIONdex particles could be used as a contrast agent in MRI (Unterweger et al., 2017). Thorat et al. studied the effect of low-Curie-temperature (TC) gadolinium-doped iron oxide nanoparticles (GdIO MNPs) in theransotic therapy. *In vitro*, these particles were employed against cancer cells by connecting them to folate and chemotherapeutic drugs. The results demonstrated strong cyto- and biohemocompatibility, as well as stable colloids with a high attraction for malignant cells. The results reveal that GdIO NPs at 1 mg mL^−1^ have no obvious hemolytic impact for up to 24 h. They lowered the viability of malignant cells by releasing chemotherapy chemicals. Furthermore, these MNPs could be employed in bioimaging using a T1–T2 dual-model magnetic resonance to better visualize cancer cells (Thorat et al., 2016). A potential candidate for MRI contrast agent could be synthetic hydroxyapatite (HAP) with outstanding biocompatibility and no inflammatory responses. Laranjeira et al. evaluated HAP’s *in vitro* cyto- and hemocompatibility with Gd^3+^/Fe^2+^/Fe^3+^/Co^2+^. The maximum magnetic moments were found in Gd and Fe(III), and Gd–HAP MNPs performed better than Fe(III) because Gd–HAP MNPs had no cytotoxicity, thrombogenicity, or hemolytic reactions, whereas Fe(III)–HAP could generate thrombogenic reactions. As a result, Gd–HAP MNPs could be employed as MRI contrast agents (Laranjeira et al., 2016). Urandur et al. studied lyotropic liquid crystalline nanostructures for breast cancer diagnostics and treatment. They loaded lyotropic liquid crystalline nanostructures (LCNs) with MnO NPs (LCN@Mn–BA) since MNPs could detect malignancies by acidosis localization and high H_2_O_2_ levels indicated oxidative stress. LCNs were also given betulinic acid (BA) as a synergistic anti-tumor agent. LCNs were shown to create radicals against malignant cells while leaving normal cells untouched. Surprisingly, LCN could slow down tumor progression with a tumor growth inhibition score of 96.5%. In comparison to the positive control, the highest concentration of LCN@Mn–BA showed 3.8%–4.3% hemolysis, indicating that the formulation of LCN can be used safely *in vivo*. Fluorescent MNPs revealed tumor localization and local anticancer response. LCNs, when combined, have the potential to be a viable therapeutic and diagnostic agent for tumor localization and treatment (Urandur et al., 2020). Microwave-induced thermoacoustic imaging (MTAI) is a technique that combines high-contrast microwave imaging with high-resolution ultrasound imaging to better diagnose deep-located malignancies. Zhang et al. used a microwave-induced thermoacoustic sensor that was activated to detect deep-seated malignancies. They created a manganous-manganic oxide-based nanoprobe (Mn_3_O_4_–PEG–RGD) that was activated by the tumor microenvironment via glutathione overexpression and acidity, resulting in enhanced conductivity via manganese ion release. Pulsed microwaves accelerated ion mobility, resulting in heating and thermoelastic expansion via the Joule effect. These modifications resulted in a strong TA wave at the tumor location. The biocompatibility of MNPs under physiological settings such as blood circulation was examined using a hemolysis test, and the results show that MNP hemolysis rates were 5% across the board. Overall, manganous-manganic oxide-based nanoprobes improved TA-signal in deep tumors and may be a viable approach for deep tumor identification (Zhang S. et al., 2022). Bevacizumab (BCZM) is a commonly used monoclonal antibody that recognizes VEGF-A, which is overexpressed during angiogenesis. Tsoukalasstudy et al. created a dual-modality nanoplatform for *in vivo* tumor vascularization imaging using single-photon computed emission tomography (SPECT) and magnetic resonance imaging (MRI). Iron oxide nanoparticles (IONPs) were coated with dimercaptosuccinic acid (DMSA) before being functionalized with the radiolabeled monoclonal antibody BCZM (Fe_3_O_4_-DMSA-SMCC-BCZM-99mTc). *In vitro* cytotoxicity tests revealed that this nanostructure has no detrimental effects on normal or cancer cells. *In vivo* biodistribution investigations in SCID mice carrying M165 tumors revealed substantial uptake in the liver, spleen, kidney, and lungs. According to the findings of this work, Fe_3_O_4_–DMSA–SMCC–BCZM–99mTc IONPs could be a valuable diagnostic tool for biomedical imaging as well as a good option for future theranostic applications in cancer (Tsoukalas et al., 2018). The goal of Karageorgou et al.’ study was to create a dual-modality PET/MR imaging probe by radiolabeling IONPs surface functionalized with the water-soluble stabilizer 2,3-dicarboxypropane-1,1-diphosphonic acid (DPD) with the positron emitter Gallium-68. *In vitro* cytotoxicity experiments revealed that normal cells were less hazardous than cancer cells. *Ex vivo* biodistribution investigations in normal Swiss mice revealed that the liver was the most uptaken, followed by the spleen. The PET pictures obtained were consistent with the *ex vivo* biodistribution data. The findings of this work suggest that Fe3O4–DPD MNPs could be a useful diagnostic tool for biomedical imaging (Karageorgou et al., 2017).

### 3.4 Hemocompatibility of MNPs in drug delivery

**FIGURE 3:**
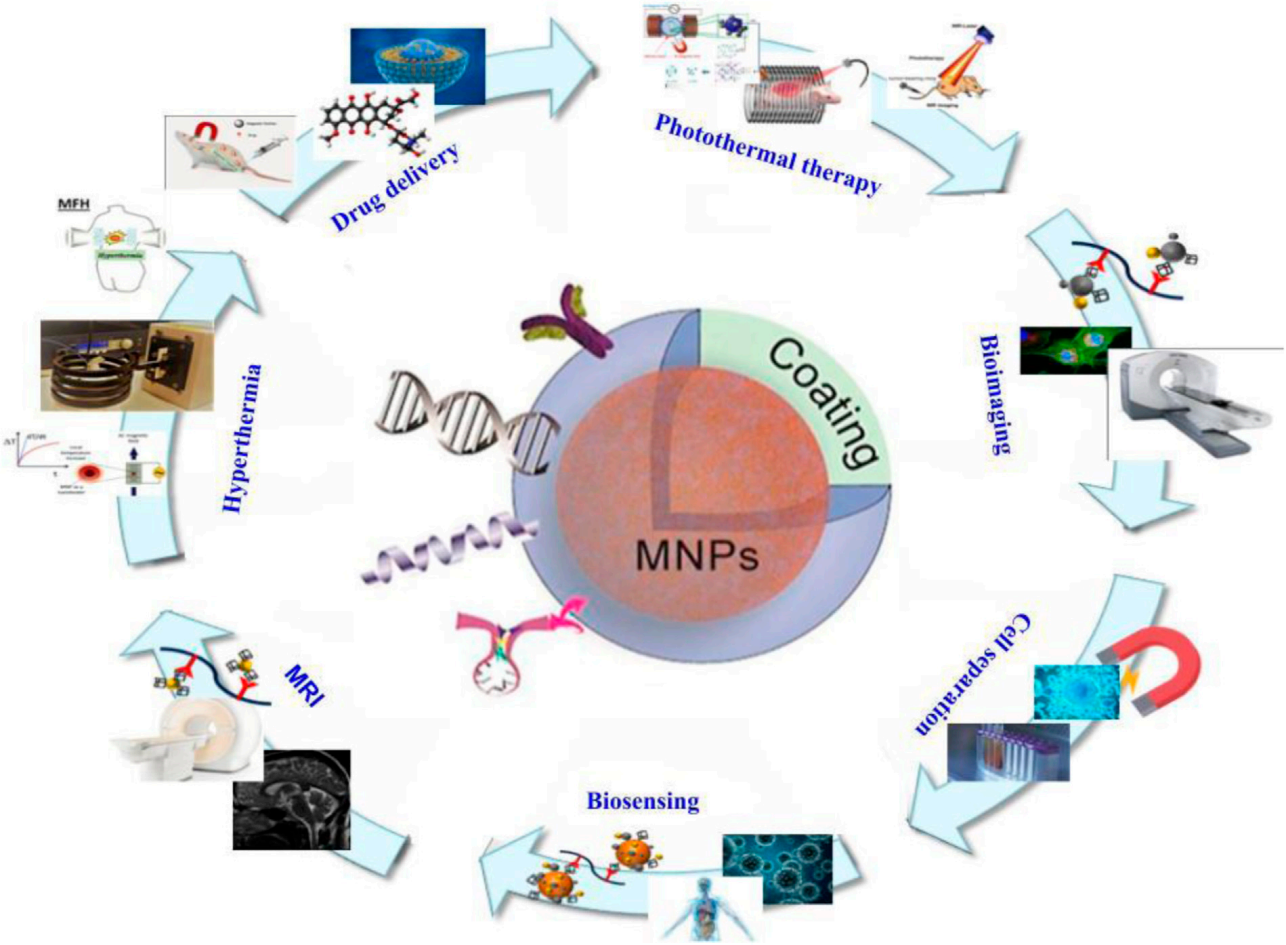
Structure of magnetic nanoparticles and their various applications in the field of medicine.

Another application for MNPs is drug delivery, not just in cancer but also in other disorders (Figure 3). These particles’ hemocompatibility is a crucial component of their use in delivering drugs in the body. Kempe et al. created MNPs for the treatment of in-stent thrombosis. These MNPs ranged in size from 10 to 30 nm, and they were salinized using tetraethyl orthosilicate with triethylene glycol and/or polyethylene glycol. Tissue plasminogen activator (tPA) was immobilized using N-hydroxysulfosuccinimide and tresyl chloride. Under all conditions tested, naked magnetite NPs and tPA-MNP conjugates were non-hemolytic. Intravenous administration of these NPs to pigs resulted in minimal hemolysis and good hemocompatibility. Furthermore, they demonstrated that tPA-nanoparticle conjugates might be employed to treat in-stent thrombosis in coronary arteries (Kempe and Kempe, 2010). Akrami et al. developed MNPs for drug delivery with high cellular uptake and extra capacity for drug loading. Fe_3_O_4_ MNPs with hydroxyapatite (HAP) and/or polyethyleneimine (PEI) functionalization and modification with cyclodextrin (CD) enhanced MNP capacity (Fe@PEI-CD and Fe@HAP-PEI-CD). Curcumin was encapsulated in these CD shells (CUR-loaded Fe@HAP-PEI-CD), resulting in pH-sensitive curcumin release. The results showed that free curcumin had less toxicity and significantly inhibited MCF-7 breast cancer cells. In the examined concentration range (10–80 mM), the maximal hemolytic percentage of Fe@PEI-CD and Fe@HAP-PEI-CD samples was 1.0% and 0.7%, respectively. Furthermore, drug absorption of curcumin loaded in MNPs was higher than that of free curcumin, and the MNPs’ good hemocompatibility made them viable agents for curcumin administration (Akrami et al., 2015). Nosrati et al. evaluated the efficiency of methotrexate (MTX)-conjugated MNPs against breast cancer cells in another investigation. They immobilized the MNPs with MTX after encapsulating them with glycine (F-Gly–MTX NPs). The average size of these MNPs was 46.82, and their cyto- and hemocompatibility were validated on HFF-2 and HEK-293 cell lines. Less than 2.4% hemolytic activity was seen at high concentrations. These compounds had a strong anticancer effect, making them promising therapeutic targets for breast cancer treatment (Nosrati et al., 2018). Attari et al. conducted a similar study in which they synthesized MNPs with arginine capping using an *in situ* and one-pot co-precipitation approach. They coupled MTX with MNPs formed by an amide bond between the amine groups on the surface of the MNPs and the carboxylic acid end groups on MTX, resulting in excellent theranostic particles (Fe–Arg–MTX). The average size of these MNPs was 27 nm, and they were likewise biocompatible. Furthermore, MTX-MNPs could be used to target several forms of cancer that have folate receptors. MTX dissociated from MNPs and induced its cytotoxic effects on cancer cells in the presence of protease-like lysosomal conditions. The modest hemolytic effect on the HRBCs was confirmed. At high concentrations, a hemolytic activity of 3.5% was detected, demonstrating HRBC biocompatibility. Collectively, these MTX-MNPs could be utilized in future cancer research studies (Attari et al., 2019). Rahimi et al. also attempted to boost the delivery of MTX to cancer cells. They created Fe_3_O_4_ nanoparticles and decorated them with biocompatible tris (2-aminoethyl) amine-functionalized nanocrystalline cellulose (AMFC@MNPs). As previously stated, the carboxylate groups of MTX molecules and amino groups of nanoparticles are conjugated together. These MNPs demonstrated strong cytotoxicity against MCF7 breast cancer, as well as pH-dependent MTX release with a high release rate at pH 5.4. Furthermore, the findings of low toxicity and hemolytic effects at high concentrations make these particles interesting therapeutic targets (Rahimi et al., 2017a). Aeineh et al. created a new curcumin delivery method based on glutathione-conjugated polyethyleneamine on the surface of Fe_3_O_4_ magnetic nanoparticles (Fe_3_O_4_–PEI–GSH–CUR). The particles had an average size of 50 nm and a larger release of curcumin in acidic environments due to their pH sensitive feature achieved via the coated polymer, as well as enhanced toxicity when compared to control NPs. The greatest hemolysis percentage for Fe_3_O_4_–PEI–GSH–CUR was 2.31%. The results of this investigation demonstrate that the designed delivery mechanism is hemocompatible and has negligible hemolytic effects. Curcumin MNPs had a 2.5-fold better serum bioavailability than free curcumin, with good biocompatibility. Furthermore, an evaluation of their MRI properties revealed that they might be employed as a contrast agent in MRI (Aeineh et al., 2018). The goal of Afzalipour et al.’s investigation was to use oncothermia and chemotherapeutic drugs against glioblastoma at the same time. Temozolomide (TMZ)-loaded MNPs conjugated with folic acid (TMZ/MNPs–FA) were utilized to treat glioma rats in the presence of an alternate magnetic field (AMF). As a result, at concentrations less than 3,000 μg/mL, both nanoparticles (MNPs–FA and TMZ/MNPs–FA) appear to be homo-compatible. The results showed that this dual strategy against cancers was highly effective, since TMZ/MNPs–FA may inhibit tumor growth and boost survival rate. As a result, MNPs and AMF could be employed to improve chemotherapy efficacy (Afzalipour et al., 2021). Feuser et al. coated MNPs with oleic acid and loaded them with mini emulsion polymerized poly (methyl methacrylate) (MNPs–PMMA) to create a hyperthermia carrier system. These NPs were 99 nm in size on average. MNPs–PMMA treatment did not cause hemolysis of RBCs, indicating that these systems do not have hemolytic capacity even at high concentrations. These findings show that these nanoparticle formulations have a high blood biocompatibility, making them a viable option for transporting medications injected intravenously. Treatment of U87MG cells with MNPs–PMMA, followed by the application of an AC magnetic field, may reduce tumor size. These NPs could be employed for further research due to their great blood compatibility (Feuser et al., 2015). Manju et al. investigated an approach for increasing MNPs’ hydrophobic drug capacity. Layer-by-layer assembly (LbL) was employed in conjugation with curcumin, a medication with low water solubility and instability. Curcumin was coupled with poly (vinylpyrroidone) (PVP-Cur) and hyaluronic acid (HA–Cur) in the LbL method to synthesize cationic and anionic curcumin and change MNPs via LbL assembly ((HA–Cur/PVP–Cur)6@MNPs). The hemolysis of (HA–Cur/PVP–Cur)6@MNPs is 0.037%, according to the results. (HA–Cur/PVP–Cur)6@MNPs were created and used to treat glioma and Caco-2 cells. Because of their increased solubility and absorption, these MNPs demonstrated enhanced cytotoxicity, cellular uptake, and effectiveness when compared to free curcumin. As a result, this technique could be utilized to improve the efficacy of hydrophobic drugs (Manju and Sreenivasan, 2011). Azizi et al. used a simple approach (Fe_3_O_4_@BSA–MTX) to produce and study MNPs functionalized with methotrexate (MTX)-conjugated bovine serum albumin (BSA). Their average size was 105.7. The enzyme-dependent release pattern of MTX from these particles was observed. These particles’ superior biocompatibility and cytotoxicity made them good biocompatible carriers for MTX (Azizi et al., 2020). Another study established a multidrug delivery system for chemotherapeutic drugs (doxorubicin (DOX) and MTX). Because of their strong pharmacological potential, they chose dendrimer-based NPs. In this investigation, dendritic grafted chitosan-coated MNPs (DPC@MNPs) with a pH-sensitive drug release profile were employed. DOX and MTX encapsulation had 96% and 68% effectiveness, respectively. Because of their high blood compatibility and interactions with various proteins, they are ideal for drug delivery systems. Furthermore, this drug delivery technology may lessen hazardous side effects while also improving drug cellular absorption. *In vivo* studies revealed few side effects, making these MNPs a promising therapeutic component (Rahimi et al., 2017b).

## 4 Conclusion

MNPs are promising agents for improvement of both diagnosis and treatment efficacy. However, as these agents are produced from metals they could induce acute or even chronic side effects such as hemolysis and inflammatory reactions. Studies tried to improve hemocompatibility and cytocompatibility of these NPs along with the increase of cellular uptake for better efficacy against different diseases. MNPs could be useful against cancers as they could target cancer cells exclusively based on their surface receptors. Combination of MNPs with polymers and other agents and conjugating them with different drugs not only improve their cellular uptake but also increase their specificity and hemo- and cytocompatibility and decrease their side effects. In addition, MNPs can be used as a contrast agent in imaging such as MRI to improve localization and early detection of tumors and other diseases. Further studies would be needed for better understanding of MNP mechanisms in the body and the development of a better drug delivery system and reducing hemolysis and side effects.
